# A BALB/c mouse model of *Mycobacterium abscessus* lung infection based on once-weekly cyclophosphamide administration

**DOI:** 10.1242/dmm.052310

**Published:** 2025-05-29

**Authors:** Binayak Rimal, Chandra M. Panthi, Ruth A. Howe, Gyanu Lamichhane

**Affiliations:** ^1^ Division of Infectious Diseases, Department of Medicine, School of Medicine, Johns Hopkins University, Baltimore, MD 21287, USA; ^2^ Center for Nontuberculous Mycobacteria and Bronchiectasis, Department of Medicine, School of Medicine, Johns Hopkins University, Baltimore, MD 21287, USA

**Keywords:** *Mycobacterium abscessus*, *Mycobacteroides abscessus*, Lung infection, Mouse model

## Abstract

*Mycobacterium abscessus* is a fast-growing non-tuberculous mycobacterium that can cause chronic lung disease leading to rapid decline in lung function. There are no FDA-approved therapies for this disease. To support the development of new treatments, an animal model of *M. abscessus* lung infection that is simple to implement and requires minimal resources is crucial to encourage broad adoption. We present a mouse model using the immunocompetent BALB/c strain, which is both widely available and cost effective. Since BALB/c mice naturally clear *M. abscessus* infections, immunosuppression is necessary to sustain bacterial growth in the lungs. Once-weekly intraperitoneal injections of the immunosuppressant cyclophosphamide at 250 mg/kg successfully induced proliferation of *M. abscessus* during the acute phase, followed by stabilization characteristic of chronic infection. This model demonstrated the efficacy of imipenem – an antibiotic commonly used in clinical settings – by significantly reducing bacterial burdens, mirroring their effects in human cases. However, clofazimine, which is also used to treat this disease, was bacteriostatic. This cost-effective and accessible mouse model is suitable for diverse laboratory environments and provides a valuable tool for preclinical evaluation of treatments for *M. abscessus* lung disease.

## Teaser

**Summary:** This study establishes a cost-effective BALB/c mouse model of *Mycobacterium abscessus* lung infection, enabling broad adoption for preclinical drug evaluation and facilitating the development of new treatments.

## INTRODUCTION

*Mycobacterium abscessus* (also known as *Mycobacteroides abscessus*, hereafter referred to as *Mab*), is a non-tuberculous mycobacterium that can cause chronic lung and soft tissue infections (Johansen et al., 2020). Individuals with lung comorbidities, such as bronchiectasis, chronic obstructive pulmonary disease or cystic fibrosis, or those who are immunosuppressed are at higher risk. *Mab* infections are often chronic, difficult to diagnose, and result in significant morbidity and mortality (Benwill and Wallace, 2014). The global incidence of *Mab* infections has been gradually rising (Dahl et al., 2022).

Currently, there are no FDA-approved antibiotics to treat *Mab* disease. The recommended treatment involves using multiple antibiotics that are approved for other conditions over several months (Griffith and Daley, 2022; Haworth et al., 2017; Varley and Winthrop, 2022). The treatment success rate with existing therapies is only 30−50% (Diel et al., 2017; Jarand et al., 2011; Kwak et al., 2019). Due to the difficulty of curing *Mab* infections and the significant adverse effects from prolonged antibiotic use, *Mab* has been declared a ‘clinical nightmare’ (Lopeman et al., 2019; Nessar et al., 2012). Despite this designation, there is a notable lack of laboratory tools necessary for pre-clinical investigations of *Mab* disease.

Recent investigations into *Mab* have primarily utilized *in-vitro* approaches, as is common with emerging diseases. Historically, animal models have been crucial in translating *in vitro* findings into new therapeutic tools and understanding disease pathology. By mimicking the interaction between the pathogen and the host, animal models provide more informative data than *in-vitro* systems for assessing the potential efficacy of experimental treatments in humans. Animal models offer a controlled experimental setting to evaluate specific variables, such as efficacy, dosing schedules, treatment duration, interactions with other treatments and adverse events. This reproducible testing environment accelerates the translation of basic research into tangible therapeutic tools, ultimately, improving patient outcomes.

Existing animal models of *Mab* disease have provided valuable insights into the virulence of isolates, the pathogenesis of the disease and the efficacy of therapeutic agents (Nicola et al., 2023). An ideal laboratory *Mab* animal model should closely mimic the disease's natural history in humans, allow aerosol infection with *Mab* to produce chronic lung disease, enable the administration of therapeutic agents via routes used in humans, and facilitate efficacy assessments using microbiological and clinical endpoints similar to those used in clinical settings (Dartois et al., 2024). Additionally, the model should be practical for laboratory use, with the animal species being readily available at a reasonable cost, easy to handle, and of small mass and size to allow large sample sizes without requiring excessive quantities of test treatments.

Among existing models, mice meet most of these requirements for a *Mab* animal model (Dartois et al., 2024). Although wild-type mouse strains gradually clear *Mab* infection, immune system alterations have enabled reliable infection and disease progression (Nicola et al., 2023; Ordway et al., 2008). Deleting genes encoding vital immune response effectors, such as GM-CSF and IFN-γ, permits *Mab* proliferation in mouse lungs (De Groote et al., 2014; Obregón-Henao et al., 2015). SCID and Nude mouse strains also support *Mab* proliferation and disease establishment. However, with their permanent loss of essential immune responses and physical compromises, a significant percentage of a cohort dies during infection, handling and treatment administration. This is particularly problematic for chronic *Mab* disease, which requires mice to survive until the disease is established and throughout the typically prolonged treatment duration, often lasting several weeks or months. Therefore, maintaining the physical competency of these mice is crucial to ensure they are minimally affected by procedures involving infection and treatment administration.

Recently, we developed a mouse model of *Mab* lung disease by using the immunocompetent C3HeB/FeJ mouse strain (Maggioncalda et al., 2020). As this mouse strain gradually clears *Mab* from its lungs, a low dose of dexamethasone sufficient for mild immunosuppression was used to permit *Mab* proliferation without obliterating the immune effector cells, such as the lymphocytes. This mouse model adequately recapitulated the efficacy of several antibiotics currently recommended for treating *Mab* disease (Rimal et al., 2023; Sriram et al., 2022; Story-Roller et al., 2019), and enabled the assessment of efficacy of experimental drugs and virulence of *Mab* strains (Galanis et al., 2022; Nicklas et al., 2022; Ochsner et al., 2023; Rimal et al., 2022, 2024a,b).

While this model meets most desirable attributes, a recent report has identified three specific attributes associated with this model, which – if improved – might make a mouse model based on this approach less resource intensive and, therefore, feasible in more laboratories (Dartois et al., 2024). First, daily administration of an immunosuppression agent − as required for this model − was considered resource intensive in settings where animal studies are not routinely undertaken. Second, the body mass of C3HeB/FeJ increases significantly between 10 and 14 weeks of age, which can make dosing of a treatment per unit body mass of the mouse a challenge. Third, female C3HeB/FeJ mice aged 5−6 weeks currently cost between $68 and $70 per unit, and are only available from a single commercial vendor. By contrast, a more commonly used mouse strain, such as BALB/c, costs between $25 and $29 per unit, and is available from several commercial vendors. Also, large sample sizes are often necessary to enhance statistical validity of a study. Therefore, to meet this criterion, a less expensive mouse strain is more desirable. The BALB/c strain is one of the most used mouse strains in biomedical research, is comparatively inexpensive, does not gain body mass rapidly and the body masses of members of a cohort are very similar.

To simplify the requirement for daily administration of dexamethasone in our current model, we tested the hypothesis if administration of another agent at a reduced dosing frequency can permit proliferation of *Mab* and development of lung disease. The immunosuppressant cyclophosphamide causes neutropenia and is used to render mice susceptible to several human bacterial pathogens (Lepak et al., 2017; Zuluaga et al., 2006). We tested various doses of cyclophosphamide at different dosing schedules to determine if once-weekly dosing can produce mild immunosuppression that is sufficient to permit *Mab* proliferation in the lungs and chronic disease development as seen in humans. We tested this approach with *Mab* strain ATCC 19977, the first isolate archived at the ATCC (Moore and Frerichs, 1953), and which hence has served as the default *Mab* reference strain in many laboratory studies. We also assessed if this mouse model responds to the standard-of-care medication, such as imipenem and clofazimine, and recapitulates treatment response in humans.

## RESULTS

### Study 1 – once-weekly intraperitoneal administration of 100 mg/kg cyclophosphamide is insufficient to permit *Mab* proliferation in the lungs of BALB/c mice

Intraperitoneal administration of 100–150 mg/kg cyclophosphamide in pathogen-free Swiss albino mice supports acute infection with *S. aureus, E. coli*, *B. fragilis* and *S. pneumoniae* (Zuluaga et al., 2006). In that publication, mice had been administered a loading dose of 150 mg/kg cyclophosphamide 4 days before infection, followed by a 100 mg/kg dose on the day of infection (Zuluaga et al., 2006). Based on these findings, we hypothesized that a higher loading dose of cyclophosphamide is necessary to establish a robust infection with *Mab,* as BALB/c mice gradually clear *Mab* from their lungs (Maggioncalda et al., 2020).

**Fig. 1.**
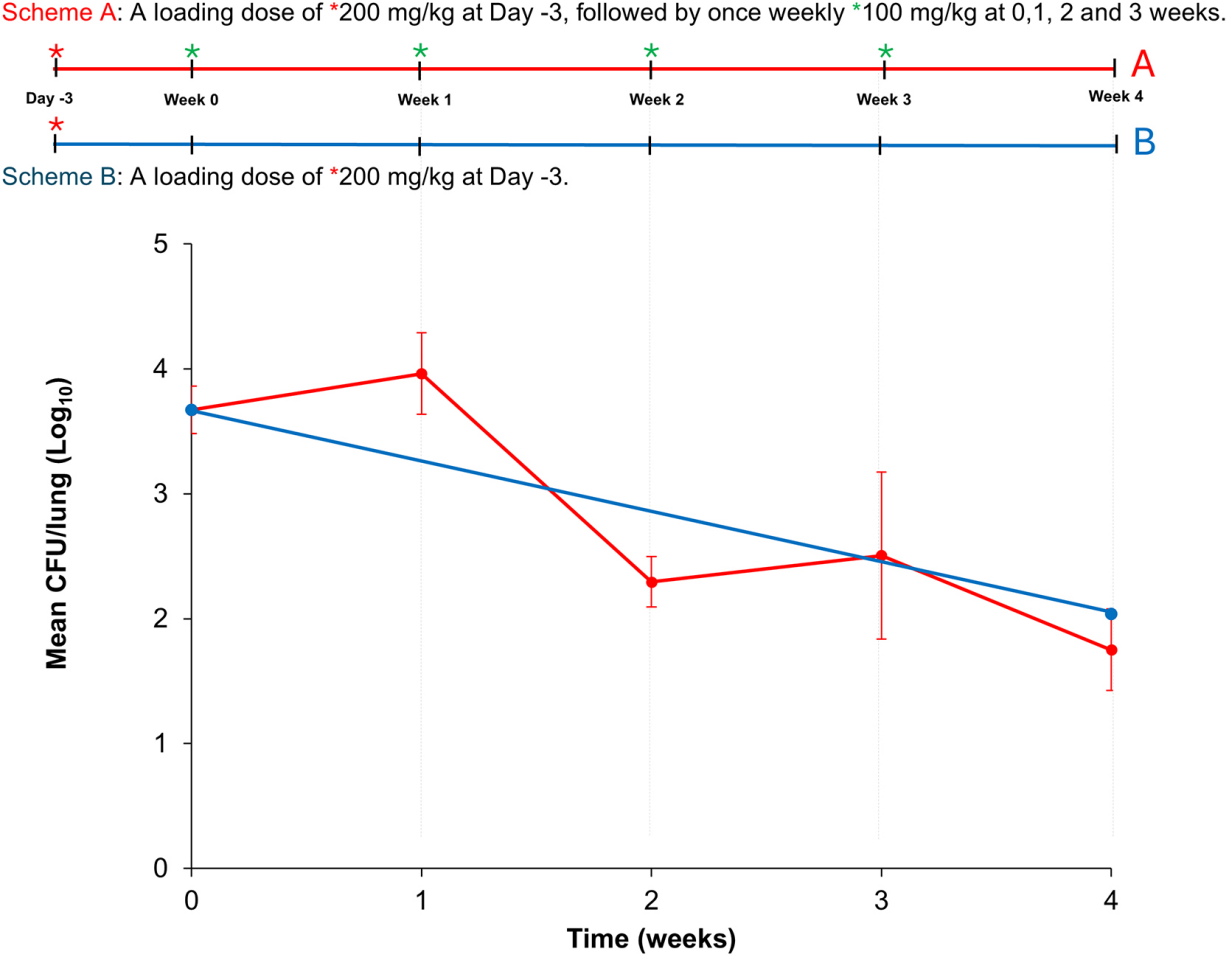
**Schematic overview of Study 1 and plot showing mean *Mab* burden in the lungs of BALB/c mice**. Top: Schematic of the experimental set-up for Study 1. All mice received a single 200 mg/kg bolus of cyclophosphamide as a loading dose 3 days before infection (Day -3) and a 100 mg/kg bolus 2 h prior infection with *M. abscessus* (*Mab*) (Week 0). After infection, mice in Group A (Scheme A; red) received a weekly dose of 100 mg/kg bolus cyclophosphamide for 3 weeks (Week 1 to Week 4), while mice in Group B (Scheme B; blue) received no further cyclophosphamide treatment. *n*=5. Bottom: Plotted is the *Mab* burden in the lungs of Group A (red) and Group B (blue) mice as the mean colony-forming unit (CFU)/lung (±s.d.) over 4 weeks. In Group A (red graph), receiving a weekly dose of 100 mg/kg cyclophosphamide, lung *Mab* burden increased by 0.3 log_10_ at the end of Week 1 but declined thereafter, leading to a net reduction of 2.0 log_10_ over the 4-week-long examination period, resulting in a paucibacillary infection. Over the same period, control Group B (blue graph) showed a similar total net reduction in lung *Mab* burden of 1.7 log_10_.

To test this hypothesis, we evaluated the effect of 200 mg/kg loading and 100 mg/kg continuation doses of cyclophosphamide in BALB/c mice, referred to here as ‘Study 1’ (Fig. 1). Three days before infection, a single bolus of 200 mg/kg cyclophosphamide was administered intraperitoneally as the loading dose. On the day of infection, a single bolus of 100 mg/kg cyclophosphamide was given intraperitoneally and 2 h later mice were infected with *Mab* strain ATCC 19977 by using the aerosol route.

We hypothesized that continued administration of cyclophosphamide is necessary to support a sustained *Mab* infection mimicking chronic infection in humans. To test this hypothesis, mice were randomly divided into two groups (A and B) after infection. Group A received a single bolus of 100 mg/kg cyclophosphamide intraperitoneally once weekly for 3 weeks. In control Group B, cyclophosphamide administration was discontinued after infection with *Mab*.

In Group A receiving the continuation dose of 100 mg/kg cyclophosphamide weekly, lung *Mab* burden increased by 0.3 log_10_ at the end of the first week but declined thereafter, leading to a net reduction of 2.0 log_10_ over the 4-week-long examination period of Study 1, resulting in a paucibacillary infection. i.e. one comprising fewer bacilli (Fig. 1). Since this infection was marked by a failure to maintain or increase the *Mab* burden − an expected characteristics of a productive infection − it did not meet the criteria for a sustained infection (Dartois et al., 2024). Similarly, at week 4 post infection, control Group B showed a 1.7 log_10_ net reduction in lung *Mab* burden. These data indicate that once-weekly administration of 100 mg/kg cyclophosphamide is insufficient to promote sustained *Mab* proliferation in the lungs of BALB/c mice.

### Study 2 – twice- but not once-weekly administration of 200 mg/kg cyclophosphamide is sufficient to permit *Mab* proliferation in the lungs of BALB/c mice

**Fig. 2.**
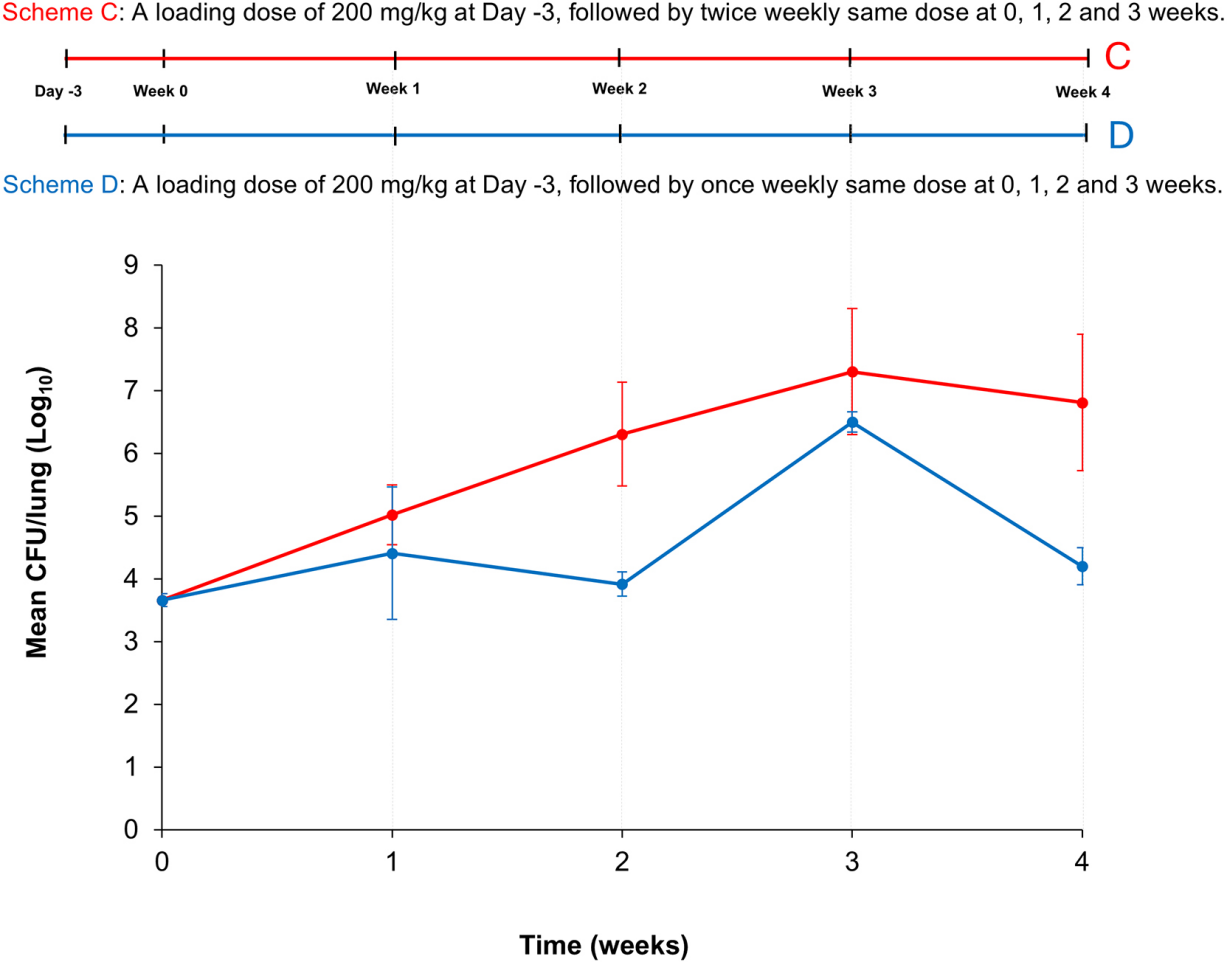
**Schematic overview of Study 2 and plot showing the mean *Mab* burden in the lungs of BALB/c mice**. Top: Schematic of the experimental set-up for Study 2. All mice received a loading dose of 200 mg/kg cyclophosphamide 3 days before infection (Day -3) and again one dose 2 h before infection with *M. abscessus* (*Mab*) (Week 0). After infection, mice in Group C received 200 mg/kg cyclophosphamide twice weekly, i.e. a total of 400 mg/kg per week, with individual doses 3.5 days apart. Mice in Group D received 200 mg/kg cyclophosphamide once weekly. *n*=5. Mean CFU/lung±s.d. is plotted. Bottom: Plotted is the *Mab* burden in the lungs of Group C (red) and D (blue) mice as the mean colony-forming unit (CFU)/lung (±s.d.) over 4 weeks. In Group C (red graph), lung *Mab* burden increased steadily each week for 3 weeks, followed by a decrease of 0.5 log_10_ CFU in Week 4, resulting in a net increase of 3.2 log_10_ CFU over the 4-week period. In Group D (blue graph), increased lung *Mab* burden at the end of Week 1 was followed by a decrease in the following week with a net increase of 0.5 log_10_ CFU over the 4-week period.

In this experimental set-up, referred to as ‘Study 2’, we hypothesized that increasing the continuation dose of cyclophosphamide permits *Mab* proliferation in mouse lungs. To test this hypothesis, mice were randomly divided into two groups (C and D) to increase both the dose and frequency of cyclophosphamide administration. Mice in both groups received 200 mg/kg cyclophosphamide 3 days before infection and 2 h before infection with *Mab* (Fig. 2). Thereafter, lung *Mab* burden was measured 1 day after infection and at the end of each week.

After infection, Group C received 200 mg/kg cyclophosphamide twice weekly, i.e. a total of 400 mg/kg per week, separated by 3.5 days. In this group, lung *Mab* burden increased steadily each week for 3 weeks but decreased 0.5 log_10_ colony-forming units (CFU) in the fourth week, resulting in a net increase of 3.2 log_10_ CFU over the 4-week period (Fig. 2).

Group D received 200 mg/kg cyclophosphamide once weekly. In this group, an increase in the lung *Mab* burden at the end of the first week was followed by a decrease in the second week, totaling to a net increase of 0.5 log_10_ CFU over 4 weeks. The inconsistent trend in mean lung *Mab* burden indicates that once-weekly administration of 200 mg/kg cyclophosphamide did not effectively support a sustained proliferation or maintenance of *Mab* burden in mouse lungs. In contrast, twice-weekly administration of 200 mg/kg cyclophosphamide supported a sustained increase in lung *Mab* burden during the first 3 weeks. None of the mice in either group died during the study period.

### Study 3 – once-weekly administration of 250 mg/kg cyclophosphamide is sufficient to permit *Mab* proliferation in the lungs of BALB/c mice

**Fig. 3.**
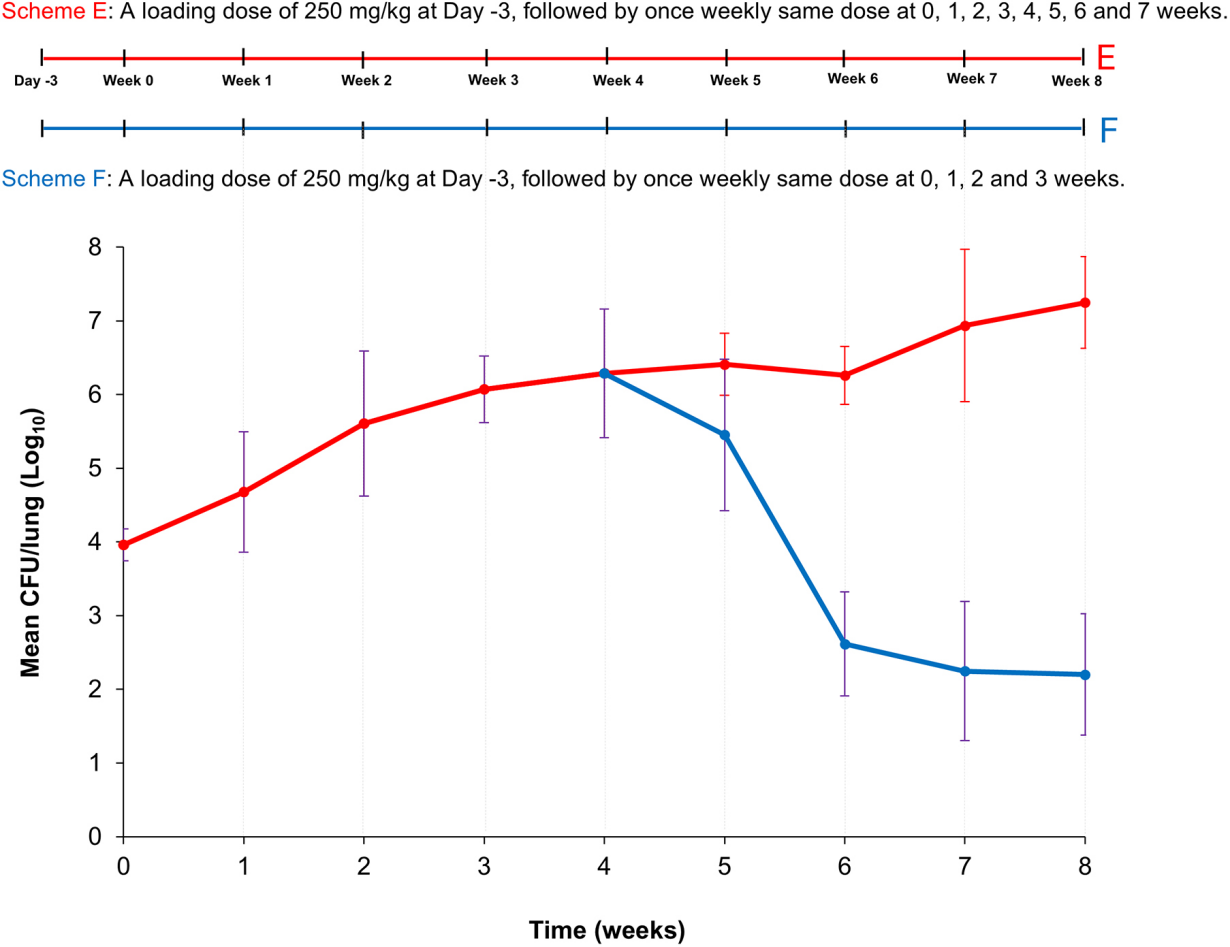
**Schematic overview of Study 3 and plot showing the mean *Mab* burden in the lungs of BALB/c mice**. Top: Schematic of the experimental set-up for Study 3. All mice received a single loading dose of 250 mg/kg cyclophosphamide 3 days before infection (Day -3) and one dose 2 h before infection with *M. abscessus* (*Mab*) (Week 0), followed by one dose once weekly for three subsequent weeks (Week 1 to Week 3). After the end of three weeks following infection, cyclophosphamide treatment was discontinued in Group F (blue). Mice in Group E (red) continued to receive a once weekly bolus of 250 mg/kg cyclophosphamide intraperitoneally. *n*=5. Mean CFU/lung±s.d. is plotted. After the end of three weeks following infection (Week 4), injection of cyclophosphamide was replaced by injection of PBS (control) in Group F (blue). Group E (red) continued to receive a once-weekly bolus of 250 mg/kg cyclophosphamide intraperitoneally until Week 8. *n*=5. Bottom: Plotted is the *Mab* burden in the lungs of Group E (red) and F (blue) mice as the mean colony-forming unit (CFU)/lung (±s.d.) over 8 weeks.

Based on the findings from Study 2, we hypothesized that increasing the cyclophosphamide dose while maintaining a once-weekly dosing schedule supports *Mab* proliferation and sustains infection in the lungs of BALB/c mice. To test this hypothesis, we evaluated the effect of once-weekly administration of 250 mg/kg cyclophosphamide on lung *Mab* burden in another experimental set-up, hereafter referred to as Study 3. We also assessed whether continuous cyclophosphamide administration is necessary to sustain *Mab* infection. Additionally, to represent the chronic phase of infection, we extended the experimentation period to 8 weeks post infection (Fig. 3).

Study 3 comprised two groups of mice (E and F), with lung *Mab* burden measured one day after infection and weekly thereafter. Both groups received a 250 mg/kg cyclophosphamide bolus 3 days before infection and 2 h before infection with *Mab* ATCC 19977 (Week 0). Following infection, all mice received 250 mg/kg cyclophosphamide once weekly for 3 weeks. At the end of 4 weeks post infection (Fig. 3, Week 4), mice were randomly divided into two equal groups (E and F). Group E continued receiving 250 mg/kg cyclophosphamide once a week for another 4 weeks (i.e. 8 weeks in total). Lung *Mab* burden in this group increased weekly and rapidly during the acute phase (first 2 weeks of infection) and more slowly during the chronic phase (beyond 4 weeks post infection), with a net increase of 3.29 log_10_ CFU over 8 weeks (Fig. 3). Group F received a final 250 mg/kg cyclophosphamide dose at the end of 3 weeks post infection, followed by intraperitoneal injection of the same volume of phosphate-buffered saline (PBS) only as a control because cyclophosphamide solution was prepared in PBS. In Group F, the lung *Mab* burden peaked 1 week after the final cyclophosphamide dose and declined thereafter, resulting in a net reduction of 4.09 log_10_ CFU from the peak burden over 4 weeks. The rapid clearance of *Mab* from the lungs after discontinuing cyclophosphamide mirrored observations from Study 1, where lung *Mab* burden decreased in mice that did not receive cyclophosphamide after the initial loading doses.

These observations indicate that discontinuation of cyclophosphamide injections does not sustain the *Mab* burden, despite a high lung *Mab* burden of >6 log_10_ CFU at the time of ceasing cyclophosphamide administration. In contrast, once-weekly intraperitoneal administration of 250 mg/kg cyclophosphamide supports an initial increase followed by maintenance of *Mab* burden in BALB/c mouse lungs. The overall aim was to identify the lowest cyclophosphamide dose necessary to promote and sustain *Mab* infection in the lungs of BALB/c mice. Study 3 demonstrated that a once-weekly intraperitoneal dose of cyclophosphamide at 250 mg/kg is sufficient to achieve this aim. To evaluate the reproducibility of these findings, we conducted an additional fourth study (Study 4) by using the same cyclophosphamide dosing regimen.

### Study 4 – the impact of 250 mg/kg cyclophosphamide, its discontinuation and efficacy of imipenem on lung *Mab* burden is reproducible

In a follow-up experimental set-up referred to as ‘Study 4’, we set three main objectives to assess: (a) whether the effect of once-weekly administration of cyclophosphamide at 250 mg/kg on BALB/c mice − as observed in Study 3 − can be reproduced in supporting *Mab* infection; (b) whether the *Mab* lung burden can be sustained over additional weeks by extending the study duration to week 10 post infection and; (c) whether this model can replicate the efficacy of imipenem, the standard-of-care drug against *Mab*, as seen in other mouse models of *Mab* lung disease (Maggioncalda et al., 2020; Story-Roller et al., 2019).

**Fig. 4.**
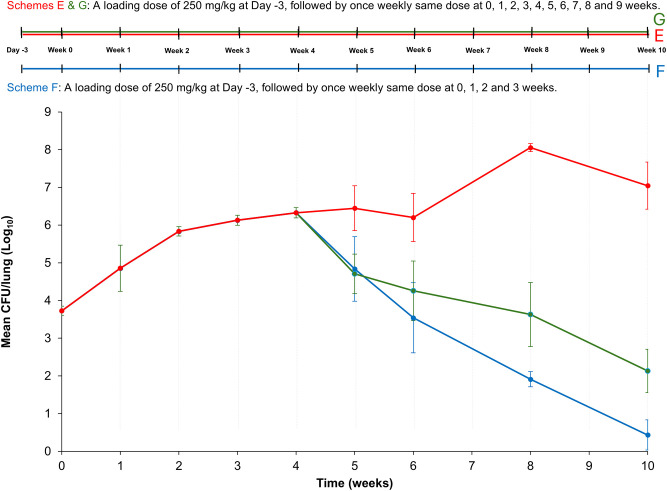
**Schematic overview of Study 4 and plot showing the mean *Mab* burden in the lungs of BALB/c mice**. Top: Schematic of the experimental set-up for Study 4. As for Study 3, all mice received a single loading dose of 250 mg/kg cyclophosphamide 3 days before infection (Day -3) and one dose 2 h before infection with *M. abscessus* (*Mab*) (Week 0), followed by one dose once weekly for three subsequent weeks (Week 1 to Week 3). After the end of three weeks following infection (Week 4), injection of cyclophosphamide was replaced by injection of PBS (control) in Group F (blue) Group E (red) continued to receive a once-weekly bolus of 250 mg/kg cyclophosphamide intraperitoneally until Week 10. At the beginning of week 4, Group G (green) received imipenem (100 mg/kg per dose) two doses per day (8–12 h apart), 7 days a week. *n*=5. Bottom: Plotted is the *Mab* burden in the lungs of Group E (red), G (green) and F (blue) as the mean colony-forming unit (CFU)/lung (±s.d.) over 10 weeks. For Group F, CFU counts were 0, 3, 0, 49 and 0. For mice with undetectable CFUs, a log_10_ CFU value of 0 was assigned. As a result, the mean log_10_ CFU of *Mab* burden is <1.

We used the same protocol as in Study 3, including the identical *Mab* isolate, mouse strain and cyclophosphamide dose, and three assessment groups (Fig. 4) − Groups E, F and G − were included in Study 4. As in Study 3, the cyclophosphamide-only group (also referred to as Group E; see Study 3) received 250 mg/kg of cyclophosphamide intraperitoneally once weekly, albeit for 10 instead of 8 weeks in total. For the cyclophosphamide discontinuation group (also referred to as Group F; see Study 3), treatment with cyclophosphamide (250 mg/kg, once weekly) was stopped after week 4 post infection as in Study 3. The cyclophosphamide + imipenem group (Group G) received the same cyclophosphamide regimen as the first group, with the addition of imipenem (100 mg/kg, twice daily) from week 4 to week 10 to evaluate its efficacy as seen in humans.

All three groups of mice were infected simultaneously by exposure to an aerosol of ATCC 19977. As in Study 3, infection in Study 4 was allowed to progress for 4 weeks to the approximate the onset of chronic disease before starting treatment with imipenem (Fig. 4). In both this study and in Study 3, the mean *Mab* lung burden at time of infection was similar − 3.73±0.12 log_10_ CFU in Study 4 and 3.96±0.22 log_10_ CFU in Study 3, with *P*=0.13 for both − indicating consistent *Mab* lung burden. Mice in Group E receiving only 250 mg/kg cyclophosphamide once weekly throughout Study 4, showed gradually increased *Mab* burden in the lung, mirroring the trend observed for Group E in Study 3. Pairwise comparisons of the mean *Mab* burden in these biological replicates (Group E) in Studies 3 and 4 at weeks 0, 1, 2, 3, 4, 5, 6 or 8 yielded *P*-values of 0.13, 0.73, 0.68, 0.82, 0.94, 0.91, 0.91 or 0.08, respectively, demonstrating consistency in *Mab* CFU burden across timepoints.

Similarly, Group F in Studies 3 and 4 represented biological replicates for which cyclophosphamide was discontinued at week 3 post infection, allowing comparison at weeks 5, 6 and 8. Pairwise comparisons of the mean *Mab* lung burden at these timepoints yielded *P*-values of 0.49, 0.16 and 0.54, respectively, supporting reproducibility regarding *Mab* burden after treatment with cyclophosphamide was discontinued.

At the final timepoint of Study 4 (10 weeks post infection), the net mean *Mab* lung burden increased by 3.31 log_10_ CFU in the Group E receiving weekly 250 mg/kg cyclophosphamide intraperitoneally. Overall, the *Mab* burden in mouse lungs increased rapidly during the acute phase and then stabilized. In Group F for which – after 4 weeks – injections of cyclophosphamide had been replaced with same-volume injections of PBS, the lung *Mab* burden peaked 1 week after the final dose of cyclophosphamide and then declined by 5.90 log_10_ CFU over 6 weeks, reaching a *Mab* lung burden of 0.43 log_10_ CFU of by the end of Study 4.

### Study 5 – efficacy of clofazimine against lung *Mab* infection in mice treated with 250 mg/kg cyclophosphamide

Clofazimine is a standard-of-care medication for *Mab* infections (Griffith and Daley, 2022; Varley and Winthrop, 2022). We, therefore, evaluated its efficacy by using the same cyclophosphamide dosing regimen as in Studies 3 and 4. The chosen dose of clofazimine – 25 mg/kg administered orally once daily – was based on the findings by Sriram and colleagues, demonstrating its effectiveness in female BALB/c mice immunosuppressed with dexamethasone. In that study, clofazimine treatment began in the second week of infection (Sriram et al., 2022). In contrast, in our Study 5, treatment commenced at the start of the fifth week of infection.

**Fig. 5.**
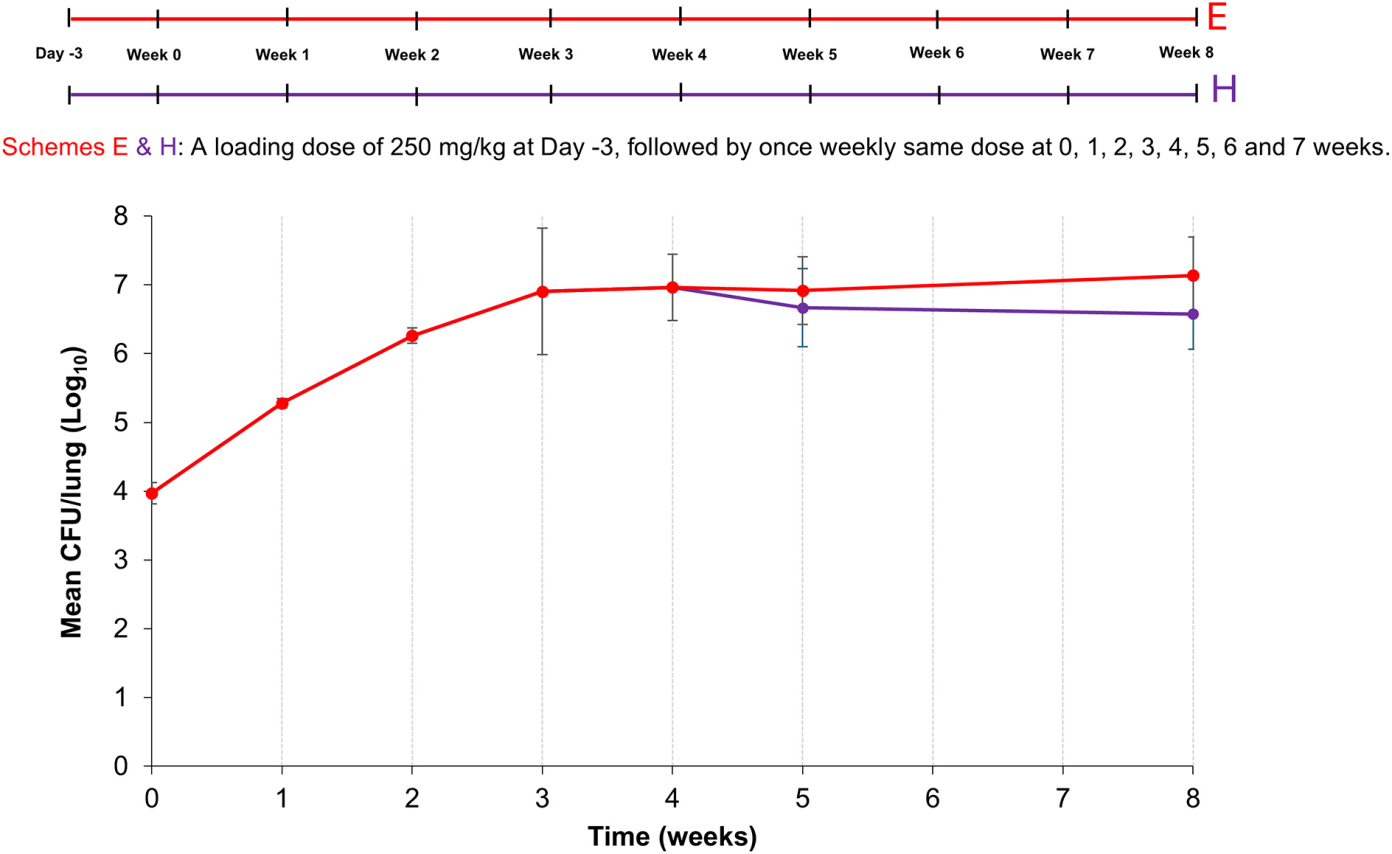
**Schematic overview of Study 5 and plot showing the mean *Mab* burden in the lungs of BALB/c mice**. Top: Schematic of the experimental set-up for Study 5. All mice received a single loading dose of 250 mg/kg cyclophosphamide 3 days before infection (Day -3) and one dose 2 h before infection with *M. abscessus* (*Mab*) (Week 0), followed by one dose once weekly for 7 subsequent weeks (Week 1 to Week 7). Groups E (red) and H (purple) continued to receive a once weekly bolus of 250 mg/kg cyclophosphamide intraperitoneally until Week 7. Group H (purple) received once daily oral 25 mg/kg clofazimine from start of week 4 until week 8. *n*=5. Bottom: Plotted is the *Mab* burden in the lungs of Group E (red) and H (purple) mice as the mean colony-forming unit (CFU)/lung (±s.d.) over 8 weeks.

In the mouse cohort receiving cyclophosphamide alone (Group E), the mean lung *Mab* burden followed a typical pattern: rapid bacterial proliferation in the initial weeks, followed by stabilization thereafter (Fig. 5). Mice treated with clofazimine showed a 0.40 log_10_ CFU reduction in lung bacterial burden after 4 weeks of treatment. At that point, clofazimine-treated mice had 0.56 log_10_ CFU fewer *Mab* in their lungs compared to untreated mice, although this difference was not statistically significant. These findings suggest that clofazimine demonstrates bacteriostatic activity in this model.

For all Studies 1–5, key indicators of illness – including rapid weight loss, hunched posture, piloerection, dehydration, lethargy, labored breathing, difficulty moving, swelling, porphyrin staining, diarrhea, eye or ear discharge, self-mutilation and changes in grooming behavior – were monitored throughout the study. None of these symptoms were observed. As outlined above, we initially used the lowest dose of cyclophosphamide, which was gradually increased to determine the minimal dose required to support *Mab* proliferation and maintenance. While higher doses of cyclophosphamide could have adversely affected mouse health, they were not necessary to achieve the objectives of our study.

## DISCUSSION

Nontuberculous mycobacterial (NTM) infections are an emerging clinical concern, particularly among patients with structural lung disease or immunosuppression. The rising incidence of these infections exceeds what would be expected from improvements in diagnostics alone (Dahl et al., 2022; Varley and Winthrop, 2022). Among NTM infections, *Mab* is the second most common pathogen and remains a major therapeutic challenge (Cristancho-Rojas et al., 2024). Current treatment regimens are lengthy, complex and often unsuccessful, highlighting the urgent need for new therapies. To support drug development, accessible and reliable preclinical animal models are essential. An ideal model should minimize costs, facility requirements and personnel time, while being readily available from multiple vendors − as recommended by a recent expert panel (Dartois et al., 2024). Such a model would not replace all others but, instead, complement them, thereby accelerating drug discovery and translational research.

Here, we present a pulmonary *Mab* infection model using BALB/c mice,  a mouse strain that is widely available and cost effective. In this model, infection was sustained through weekly intraperitoneal injections of 250 mg/kg cyclophosphamide following exposure to aerosolized *Mab*. Our model reliably produced rapid establishment of lung infection within the first 2 weeks, followed by a steady chronic increase in bacterial burden over an 8- to 10-week study period. This progression culminated in a mean 3 log_10_ increase in lung CFUs without observable attrition in mice.

The model successfully reproduced the efficacy of imipenem, a broad-spectrum antibiotic, in reducing bacterial load. This result mirrored findings from another model using daily treatment with dexamethasone for immunosuppression (Story-Roller et al., 2019). In our current study, daily treatment with clofazimine (25 mg/kg) exhibited bacteriostatic activity, as it prevented *Mab* growth but did not reduce its burden (Fig. 5). Notably, a previous study using daily treatment with the glucocorticoid dexamethasone as the immunosuppressive agent has reported different results, i.e. during the first week, clofazimine initially demonstrated bacteriostatic effects but the agent became bactericidal in the subsequent weeks (Sriram et al., 2022). Several key experimental conditions were identical between our current study and that by Sriram et al., including the mouse strain, *Mab* strain, and the dose and dosing frequency of clofazimine treatment. However, two crucial differences are likely to have contributed to the observed variations in drug efficacy. First, the immunosuppressive agent: Sriram and colleagues had used daily subcutaneous injections of 5 mg/kg dexamethasone (Sriram et al., 2022), while our current study used weekly intraperitoneal injections of 250 mg/kg cyclophosphamide. Second, the treatment timing: clofazimine treatment began in the second week of infection in the previous study but was delayed until the fifth week in the current study. Interestingly, despite these differences, imipenem showed comparable efficacy in both models. This suggests that the stage of infection when treatment begins has a greater impact on clofazimine efficacy than initially anticipated. The different effects of cyclophosphamide and dexamethasone on the immune system may influence the varying efficacy of clofazimine in the two models. Additionally, it is not known whether cyclophosphamide interacts with clofazimine, having an impact on its efficacy. Since these factors were beyond the scope of this study, future research focusing on how cyclophosphamide impacts the pharmacokinetics of drugs should be explored.

The use of cyclophosphamide to promote *Mab* growth in the lungs of BALB/c mice has been described in two previous publications. In the first, by Zhang and colleagues, mice received an intraperitoneal injection of 150 mg/kg cyclophosphamide 4 and days before intranasal *Mab* infection, followed by antibiotic treatment on day 3 after infection (Zhang et al., 2020). In the second, Sun et al. treated mice with 100 mg/kg cyclophosphamide intraperitoneally 1 day before and 4 days post infection intranasally, followed by 75 mg/kg doses on the days 8 and 12 post infection (Sun et al., 2024). Although the latter studies and our current one used the same mouse strain, animals of the same sex and age, and administration the immunosuppressant cyclophosphamide via the intraperitoneal route were the same, key differences exist. First, both earlier studies used intranasal inoculation of *Mab* suspension to infect mice, whereas we used aerosolization, allowing mice to naturally inhale aerosolized *Mab*. Second, in our current study duration of infection was allowed to progress before starting therapeutic treatments. Third, it unclear whether all three studies used the same strain of *Mab.* Therefore, variations between findings of our study and of the previous two are likely to stem from these differences in experimental design.

A limitation of our study is the potential impact of cyclophosphamide on the immune system of the host, which may influence its overall efficacy. Cyclophosphamide exerts its immunosuppressive effects by targeting rapidly proliferating immune cells, leading to a reduction in lymphocyte numbers and a depressed response to mitogens while also promoting specific effector T-cells (Nakahara et al., 2010; Winkelstein et al., 1972; Xu and Zhang, 2015). This mechanism differs from that triggered by dexamethasone – the latter inhibits immune cell proliferation without directly causing cell death. These distinct immunosuppressive pathways could differentially affect host−pathogen interactions and, consequently, the efficacy of therapeutic agents. Immunopathology studies to assess these effects were beyond the scope of the current stage of model development and were, therefore, not included. As a result, no claims relevant to pathology have been made here. However, we plan to incorporate immunopathological assessments in future studies to provide a more comprehensive understanding of the model and its implications for drug efficacy.

Our current study primarily focused on pulmonary infection and did not include gross or histopathological analyses. Such assessments may be important for some researchers, and are likely to vary depending on the specific *Mab* strain used and other factors intrinsic to the model. We selected *Mab* strain ATCC 19977, which is commonly used as laboratory reference for *Mab* to provide a consistent baseline for most laboratories. However, we did not evaluate extrapulmonary dissemination, which may be influenced by both the mouse strain and the immunosuppressive agent. When adapting the BALB/c+cyclophosphamide model for other *Mab* strains, particularly clinical isolates, researchers might need to adjust the cyclophosphamide dosing to maintain infection through chronic phases. Future studies are needed to refine this model for broader applications, enabling it to support the development of new treatments for *Mab* infections.

## MATERIALS AND METHODS

### Ethics

Mouse handling and procedures were undertaken in adherence to the national and Johns Hopkins University Animal Care and Use Committee guidelines approved by the Johns Hopkins University Animal Care and Use Committee (animal protocol number MO23M163).

### Bacterial strains and growth conditions

*Mycobacterium abscessus* (*Mab*) strain ATCC 19977, first isolated in 1950 (Moore and Frerichs, 1953) and commonly used in laboratory studies as a reference for *Mab*, was procured from ATCC and used in all studies. For infecting mice, ATCC 19977 was grown in Middlebrook 7H9 broth (Difco, catalog no. 271310) supplemented with 0.5% glycerol, 0.05% Tween-80 and 10% albumin−dextrose−sodium chloride enrichment as described (Larsen, 2000), in an orbital shaker at 220 rpm at 37°C. *Mab* in mouse lungs was recovered by culturing lung homogenates on Middlebrook 7H11 selective agar (Difco, catalog no. 283810) supplemented with 0.5% glycerol, 10% albumin–dextrose–sodium chloride enrichment, 50 μg/ml cycloheximide (Sigma-Aldrich, catalog no. C7698), and 50 μg/ml carbenicillin (Research Products International, catalog no. C46000).

### Mouse infections

For all studies, 4- to 5-week-old female BALB/c mice were procured and housed in a biosafety level 2 animal vivarium. Each study was conducted at different times with separate batches of mice. For example, mice for Study 1 were obtained on February 15, 2023, while those for Study 5 were obtained on February 13, 2024. Following arrival in our vivarium, mice were allowed to acclimatize for 7−14 days prior to initiating the studies. For Studies 1, 2, 3 and 4, mice were procured from the Jackson Laboratory (Bar Harbor, ME, USA) and for Study 5, mice were procured from the Charles River Laboratory (Wilmington, MA, USA) to assess reproducibility of the model with mice procured from distinct vendors.

To infect mice, a fresh *Mab* culture at exponential phase A_600nm_=1.00−1.20 was diluted in Middlebrook 7H9 broth to A_600nm_=0.1. Of this suspension 10 ml was aerosolized with a nebulizer attached to Glas-Col Inhalation Exposure System A4212 (Glas-Col, Terre Haute, IN, USA) into a chamber that houses mice, with the infection sequence comprising 15 min of pre-heat, 30 min of *Mab* suspension aerosolization into the chamber, 30 min of aerosol decay, and 15 min of surface decontamination with ultraviolet light.

To determine *Mab* implantation in the lungs, five mice were sacrificed 1 day post infection (designated ‘week 0’) and their lungs aseptically extracted, homogenized in phosphate-buffered saline (PBS) pH 7.4, with 2 mm glass beads by bead-beating for 30 s at 4000 rpm (Minilys, Bertin Instruments), 0.1 ml of appropriate tenfold dilutions were inoculated onto Middlebrook 7H11 agar, incubated at 30°C for 7 days and *Mab* colony forming units were enumerated. In each study, five mice per time point per group were allocated and their *Mab* burden in the lungs determined. If no colony-forming units (CFUs) were detected in the initial inoculation, the remaining homogenate was plated onto Middlebrook 7H11 agar and incubated at 30°C for 7 days. CFUs were then enumerated. Consequently, the detection limit for *Mab* in lung homogenates was determined to be <10 CFUs. For data analysis, the bacterial burden of each mouse was first log_10_ transformed. The mean of these log_10_-transformed CFU values was then calculated and plotted in Figs 1–5. In Study 4, Group F, no CFUs were recovered from three of the five mice after plating their entire lung homogenate, so their log_10_ CFU burden was recorded as 0. For the remaining two mice, 3 and 49 CFUs (log10=0.48 and CFUs (log10=1.69, respectively) were recovered. The mean of these five log_10_ CFU values is 0.43. Therefore, the log_10_ CFU burden for Group F, as shown in Fig. 4, is reported as <1.

### Cyclophosphamide preparation and administration

Cyclophosphamide solution was prepared by dissolving cyclophosphamide powder (Sigma-Aldrich, C0768) in sterile PBS pH 7.4 (Quality Biologicals, 114-058-101). Fresh cyclophosphamide solution was prepared prior to each administration and a 0.2 ml bolus of this solution was administered intraperitoneally to each mouse by using a 1-ml syringe (Becton & Dickinson, 309659) fitted with 0.27-gauge needle (Becton & Dickinson, 305109). The first dose of cyclophosphamide was administered 3 days prior to *Mab* infection. The second dose was administered ∼2 h prior to infecting mice. Subsequent cyclophosphamide dosing schedules for each study are described in under ‘Results’ and shown in corresponding figures. As the average body mass per mouse was ∼20 g, cyclophosphamide solutions were prepared at concentrations that would deliver the intended dose in a 0.2 ml bolus. For instance, to deliver a 250 mg/kg dose, 25 mg/ml cyclophosphamide solution was prepared by dissolving powdered cyclophosphamide (Sigma-Aldrich, C0768) in 1×PBS and 0.2 ml bolus of this solution was administered to each mouse.

### Imipenem and clofazimine preparations and administration

Imipenem was administered twice daily via subcutaneous injection of 0.2 ml bolus of 10 mg/ml per dose. At the beginning of Study 4 procedures in which imipenem was administered, the daily dose of imipenem powder (CAS no. 74431-23-5, Octagon Chemicals Limited) was weighed out into polypropylene tubes and stored at −20°C. Each day, the designated tube was retrieved, and sterile 1×PBS (pH 7.4) was added to achieve a concentration of 10 mg/ml. The suspension was vortexed and then sonicated with a Sonic Dismembrator (Fisher Scientific, Model 100) at 50% power for 15 s per cycle, using 1−2 cycles, until a clear hazel-colored solution had formed. Of this solution, a 0.2 ml bolus was administered by subcutaneous injection using a 1-ml syringe (Becton & Dickinson, 309659) fitted with 0.27-gauge needle (Becton & Dickinson, 305109).

For clofazimine (Sigma-Aldrich, C8895), the weekly amount of powder was weighed into 50 ml polypropylene tubes and stored at 4°C. At the beginning of each week, the weekly aliquot was retrieved, mixed with 0.05% agarose to reach a concentration of 2.5 mg/ml and vortexed for 5 min. This suspension was then sonicated at 50% power for 15 s per cycle, with 2−3 cycles, until a matte red and opaque homogeneous colloidal suspension was achieved. Aliquots necessary for each day were transferred to tubes and stored at 4°C. Of this suspension, a 0.2 ml bolus was orally administered to each mouse once daily using an oral gavage needle fitted to a 1 ml syringe (Becton & Dickinson, 309659).

### Data analysis

Raw lung CFU data were analyzed and the mean±s.d. calculated for each group at each timepoint. Results are shown as dot plots in Figs 1–5. To assess the variance between treatment groups at each timepoint, one-way ANOVA multi comparison was performed.
